# Supplementary material for: Pre-analytical variables influence zinc measurement in blood samples
Source: PLoS One. 2023 Sep 15;18(9):e0286073. doi: 10.1371/journal.pone.0286073 (PMC10503700; doi:10.1371/journal.pone.0286073)

S1 Fig. Zinc concentrations in venous samples do not differ by blood collection tube (BCT) manufacturer. Correlation plots comparing circulating zinc values from **(A)** venous plasma from BD and Sarstedt BCTs and **(C)** venous serum from BD and Sarstedt BCTs are shown. Each circle represents the zinc level (mean ± SD, *n*=1-3) for an individual participant, with linear regression and 95% confidence interval indicated by a solid red and dotted red line, respectively. The line of concordance is shown as a solid black line for comparison. Since there was no significant difference in the mean zinc concentrations for either matrix, Bland-Altman plots are only shown for illustrative purposes for **(B)** venous plasma and **(D)** venous serum samples. No significant bias was measured. Each circle represents the zinc level for an individual participant, with average distance and 95% confidence interval indicated by a solid red and dotted red line, respectively.

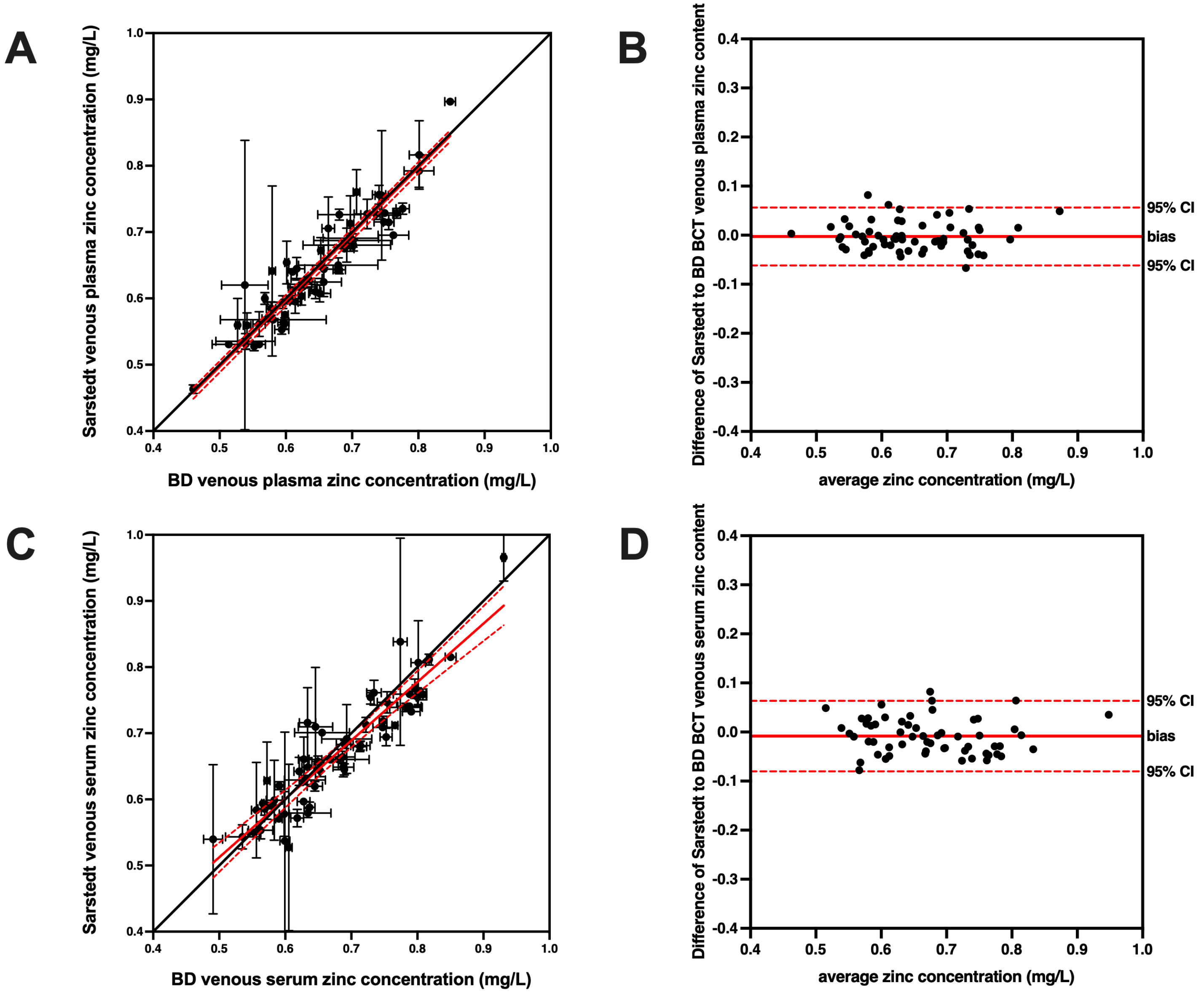

Supplement: S1 Fig — Correlation plots comparing circulating zinc values from (A) venous plasma from BD and Sarstedt BCTs and (C) venous serum from BD and Sarstedt BCTs are shown. Each circle represents the zinc level (mean ± SD, n = 1–3) for an individual participant, with linear regression and 95% confidence interval indicated by a solid red and dotted red line, respectively. The line of concordance is shown as a solid black line for comparison. Since there was no significant difference in the mean zinc concentrations for either matrix, Bland-Altman plots are only shown for illustrative purposes for (B) venous plasma and (D) venous serum samples. No significant bias was measured. Each circle represents the zinc level for an individual participant, with average distance and 95% confidence interval indicated by a solid red and dotted red line, respectively. (PDF) [file pone.0286073.s001.pdf]
