# Supplementary material for: Pre-analytical variables influence zinc measurement in blood samples
Source: PLoS One. 2023 Sep 15;18(9):e0286073. doi: 10.1371/journal.pone.0286073 (PMC10503700; doi:10.1371/journal.pone.0286073)
Supplement: S1 Table — Hemolysis is indicated by hemoglobin content (mean in mg/dL, n = 1–3) for all plasma or serum samples at all blood draw sites, blood matrices, blood collection tubes manufacturers, processing delay, and holding temperatures are shown. Samples processed immediately are marked t = 0 and temp = NA. Processing delay is indicated for 4 hour (t = 4) or 24 hours (t = 24). Holding temperature is indicated for 4°C (temp = 4C), 20°C (temp = 20C), or 37°C (temp = 37C). Missing values due to lack of blood volume are identified with an ‘NA’. Hemoglobin values greater than 100 mg/dL (elevated hemolysis) are identified with red font. (PDF) [file pone.0286073.s002.pdf]

S1 Table. Hemolysis levels in all study participant samples. Hemolysis is indicated by hemoglobin content (mean in mg/dL, *n*=1-3) for all plasma or serum samples at all blood draw sites, blood matrices, blood collection tubes manufacturers, processing delay, and holding temperatures are shown. Samples processed immediately are marked t=0 and temp=NA. Processing delay is indicated for 4 hour (t=4) or 24 hours (t=24). Holding temperature is indicated for 4°C (temp=4C), 20°C (temp=20C), or 37°C (temp=37C). Missing values due to lack of blood volume are identified with an 'NA'. Hemoglobin values greater than 100 mg/dL (elevated hemolysis) are identified with red font.

participant #	capillary	venous	venous	capillary	venous	venous	venous	venous	venous	venous	venous	venous	venous	venous	venous	venous	venous	venous
	BD	BD	BD	Sarstedt	Sarstedt	Sarstedt	BD	BD	BD	BD	BD	BD	BD	BD	BD	BD	BD	BD
	plasma	plasma	serum	plasma	plasma	serum	plasma	plasma	plasma	serum	serum	serum	plasma	plasma	plasma	serum	serum	serum
	t=0	t=0	t=0	t=0	t=0	t=0	t=4	t=4	t=4	t=4	t=4	t=4	t=24	t=24	t=24	t=24	t=24	t=24
	temp=NA	temp=NA	temp=NA	temp=NA	temp=NA	temp=NA	temp=4C	temp=22C	temp=37C	temp=4C	temp=22C	temp=37C	temp=4C	temp=22C	temp=37C	temp=4C	temp=22C	temp=37C
1	NA	7.3	0.4	NA	0.0	0.0	4.1	15.0	14.6	0.0	30.6	0.0	0.0	0.0	0.0	NA	NA	25.0
2	NA	13.2	9.9	NA	1.0	0.0	11.5	NA	39.4	NA	18.6	37.0	42.2	23.3	42.1	NA	NA	30.9
3	NA	0.3	8.5	NA	0.0	0.0	0.0	4.0	11.8	9.6	0.0	6.7	NA	0.0	14.2	0.0	0.0	0.0
4	14.7	16.4	21.2	NA	5.7	0.0	19.9	13.6	30.9	0.9	0.4	24.6	30.0	19.8	26.7	NA	0.4	5.3
5	NA	8.8	4.0	NA	0.0	0.0	9.1	58.4	8.0	0.0	NA	2.0	17.0	13.5	21.5	1.7	0.0	0.0
6	NA	0.0	0.0	NA	0.0	0.0	NA	NA	NA	0.0	0.0	0.0	NA	NA	NA	0.0	0.0	0.0
7	NA	0.0	21.1	NA	3.4	45.0	0.0	NA	2.9	0.0	0.0	0.0	12.4	22.7	19.6	0.0	0.0	0.0
8	NA	0.0	0.0	NA	0.0	0.0	0.0	0.0	8.5	0.0	0.0	0.0	0.0	0.0	0.0	1.3	0.0	0.0
9	54.4	11.7	11.5	NA	5.3	0.0	30.0	22.3	32.4	2.9	8.5	14.3	33.2	21.2	25.6	4.0	3.1	5.3
10	NA	0.0	0.0	NA	0.0	0.0	0.0	0.0	4.7	0.0	0.0	2.3	2.1	0.0	0.0	0.0	0.0	0.0
11	0.0	7.0	6.8	NA	0.0	0.0	1.3	9.7	16.8	0.0	0.0	0.0	15.6	8.0	12.8	0.0	0.0	1.0
12	NA	49.8	46.9	NA	32.2	10.0	30.3	41.7	62.0	16.1	NA	37.2	54.4	63.9	NA	NA	NA	NA
13	2.2	2.2	0.0	NA	0.0	0.0	5.2	6.2	12.9	0.0	0.0	0.7	9.5	12.6	20.3	0.0	0.0	0.0
14	NA	0.0	0.0	NA	0.0	0.0	2.9	0.0	4.3	NA	NA	2.9	9.8	13.8	14.0	NA	NA	NA
15	0.0	10.8	0.0	NA	0.0	0.0	1.8	0.0	12.8	0.0	0.0	0.8	8.1	13.9	33.6	15.1	0.0	0.0
16	0.0	5.5	11.4	NA	0.0	0.0	1.5	3.7	14.3	0.0	0.0	0.0	6.1	0.0	7.6	21.9	17.7	11.2
17	26.8	24.2	30.7	NA	16.6	6.2	19.3	27.7	41.2	6.7	13.6	30.1	27.7	NA	27.0	50.4	40.1	39.3
18	NA	6.8	19.4	NA	0.0	0.0	5.2	8.3	18.8	0.0	0.0	18.8	21.3	19.1	49.3	0.0	0.0	0.0
19	15.0	0.0	29.4	NA	4.4	0.0	0.0	2.0	12.3	0.0	0.0	1.5	9.7	8.2	19.7	0.0	0.0	4.5
20	40.9	0.0	3.7	NA	0.0	9.4	28.4	31.0	39.4	1.0	NA	25.7	54.7	50.8	58.0	24.8	3.7	33.9
21	16.1	39.5	17.7	NA	0.0	0.0	4.2	1.1	13.5	0.0	0.0	14.7	13.5	8.7	15.6	0.0	0.6	13.9
22	NA	7.5	115.3	NA	0.0	0.0	2.7	9.7	28.5	0.0	0.0	12.1	15.4	24.3	34.1	NA	NA	NA
23	10.4	4.7	30.1	NA	0.0	0.0	9.6	13.2	21.3	0.0	0.0	11.6	16.1	15.0	28.5	11.3	9.6	9.2
24	15.3	11.1	24.4	NA	4.1	0.0	9.6	14.1	31.0	0.0	0.0	10.9	32.9	21.9	39.2	8.4	8.6	21.3
25	NA	0.0	NA	NA	NA	0.0	0.0	0.0	7.9	0.0	0.0	0.0	9.5	7.3	3.2	0.0	0.0	0.0
26	34.3	19.1	20.7	NA	10.0	0.0	17.4	18.5	24.3	2.4	3.8	14.1	27.9	25.5	23.9	15.8	5.1	19.2
27	NA	0.0	14.6	0.0	0.0	0.0	1.6	1.0	12.7	0.0	NA	8.6	12.0	25.0	15.3	3.9	0.0	9.2
28	NA	1.8	15.9	NA	0.0	0.0	NA	NA	5.7	0.0	NA	3.4	19.1	9.5	21.0	0.0	0.0	1.4

**29**	0.0	0.0	0.0	NA	0.0	0.0	0.0	0.0	0.0	0.0	0.0	0.0	6.2	0.0	NA	NA	0.0	6.1
**30**	3.4	0.0	0.3	NA	NA	0.0	0.0	3.9	4.1	0.0	0.0	0.0	9.1	7.6	16.7	0.2	0.0	1.7
**31**	8.3	9.3	17.6	NA	2.1	0.0	14.7	12.8	27.1	0.0	5.0	12.9	24.8	15.6	22.8	5.8	13.3	22.9
**32**	0.0	0.0	0.0	NA	0.0	0.0	0.0	0.0	0.0	0.0	0.0	0.0	0.0	0.0	0.0	0.0	0.0	0.0
**33**	NA	27.2	45.1	NA	27.5	11.4	27.2	45.1	27.5	11.4	27.2	45.1	27.5	11.4	27.2	NA	NA	NA
**34**	0.0	0.0	55.5	NA	32.3	6.1	26.3	40.8	63.4	0.0	13.4	53.1	23.8	18.5	24.6	0.0	NA	NA
**35**	NA	16.2	25.2	NA	2.0	0.0	8.3	10.2	16.4	0.0	0.4	12.2	18.4	18.2	26.5	15.8	5.6	13.7
**36**	0.6	15.7	19.0	NA	0.0	0.0	NA	2.8	20.4	0.0	0.0	2.3	17.1	18.6	33.2	7.8	0.0	3.4
**37**	NA	15.4	38.0	NA	0.0	0.0	21.2	11.8	11.0	6.9	0.0	0.0	45.3	9.6	33.1	0.0	NA	17.6
**38**	0.0	0.0	16.5	NA	0.0	0.0	0.0	0.0	3.4	0.0	0.0	0.0	0.6	8.0	11.9	0.0	NA	NA
**39**	NA	0.0	4.0	NA	0.0	0.0	0.0	0.0	0.0	0.0	0.0	0.0	4.1	23.0	34.1	0.0	NA	0.4
**40**	NA	0.0	0.0	NA	0.0	0.0	0.0	0.0	0.0	0.0	0.0	0.0	7.0	10.0	19.9	0.0	0.0	0.0
**41**	NA	0.0	0.0	15.1	0.0	0.0	NA	NA	NA	0.0	0.0	0.0	NA	NA	NA	0.0	0.0	0.0
**42**	0.0	0.0	1.0	NA	0.0	0.0	0.0	0.0	0.1	0.0	0.0	0.0	0.0	0.0	0.0	0.0	0.0	0.0
**43**	230.1	0.0	3.4	NA	0.0	0.0	2.0	0.0	0.0	0.0	0.0	0.0	9.7	1.1	0.0	0.0	NA	0.0
**44**	27.6	0.0	31.5	0.0	0.0	0.0	1.1	1.3	13.2	0.0	NA	0.0	NA	9.3	NA	0.0	0.0	0.0
**45**	NA	0.0	4.7	NA	0.0	0.0	0.0	0.0	0.0	0.0	9.8	0.0	0.0	0.0	3.1	0.0	0.0	0.0
**46**	NA	12.5	0.0	NA	16.8	0.0	0.0	0.0	1.8	0.0	0.0	0.0	0.0	0.0	0.0	0.0	0.1	0.0
**47**	NA	0.0	0.0	NA	9.0	0.0	2.6	0.0	0.0	0.0	0.0	0.0	NA	NA	NA	4.0	0.0	0.0
**48**	NA	NA	0.0	NA	24.3	0.0	7.3	12.3	13.2	0.0	0.0	0.6	11.5	20.1	13.7	5.0	0.0	2.2
**49**	6.1	0.0	0.0	NA	11.7	0.0	4.9	0.0	3.2	0.0	0.0	1.1	14.9	19.9	19.7	7.6	0.0	8.7
**50**	21.1	18.4	13.4	NA	41.7	9.0	38.4	29.7	34.1	20.5	21.3	31.4	29.4	31.3	36.5	28.0	14.4	27.3
**51**	0.0	6.7	0.0	45.4	24.8	0.0	15.1	11.1	11.8	0.0	0.0	9.7	19.5	16.8	22.8	17.2	9.6	21.1
**52**	16.6	19.2	17.7	48.1	40.2	5.6	22.3	23.0	29.9	10.6	20.3	27.0	16.9	10.1	16.1	0.0	0.0	4.0
**53**	0.0	27.1	0.0	NA	5.9	0.0	NA	NA	NA	0.0	0.0	5.9	NA	NA	NA	8.4	0.0	17.2
**54**	12.9	12.5	1.9	19.5	34.2	0.0	12.7	26.5	23.3	3.8	5.5	12.0	19.7	6.6	11.7	16.8	16.8	23.9
**55**	NA	4.5	23.1	NA	6.7	0.0	5.3	10.5	15.8	0.0	0.0	5.9	20.7	11.5	16.1	6.8	0.0	14.5
**56**	8.3	0.0	0.0	NA	9.7	0.0	0.0	0.0	6.1	0.0	0.0	0.0	4.0	5.3	13.6	1.8	0.0	8.3
**57**	4.0	8.2	0.0	NA	6.9	0.0	0.0	0.0	4.6	6.0	3.9	4.7	12.1	8.0	15.9	6.7	0.0	2.2
**58**	NA	0.0	0.0	NA	0.0	0.0	0.0	0.0	0.0	0.0	0.0	0.0	0.0	0.0	0.7	0.0	0.0	0.0
**59**	0.5	0.5	0.4	NA	0.6	0.4	0.5	0.5	0.5	0.4	0.4	0.5	0.5	0.5	0.5	0.5	0.5	0.5
**60**	25.5	7.0	0.0	NA	33.5	0.0	0.5	0.5	0.5	0.4	0.4	0.5	0.5	0.5	0.6	0.5	0.5	0.5
