# Supplementary material for: Pre-analytical variables influence zinc measurement in blood samples
Source: PLoS One. 2023 Sep 15;18(9):e0286073. doi: 10.1371/journal.pone.0286073 (PMC10503700; doi:10.1371/journal.pone.0286073)
Supplement: S2 Table — The zinc measurements for participants for all blood draw sites, blood matrices, blood collection tubes manufacturers, processing delay, and holding temperatures are shown. Triplicate values (in mg/L) are shown for all plasma or serum samples. Samples processed immediately are marked t = 0 and temp = NA. Processing delay is indicated for 4 hour (t = 4) or 24 hours (t = 24). Holding temperature is indicated for 4°C (temp = 4C), 20°C (temp = 20C), or 37°C (temp = 37C). Missing values due to lack of blood volume are identified with an ‘NA’. (PDF) [file pone.0286073.s003.pdf]

S2 Table. Zinc values in all study participant samples. The zinc measurements for participants for all blood draw sites, blood matrices, blood collection tubes manufacturers, processing delay, and holding temperatures are shown. Triplicate values (in mg/L) are shown for all plasma or serum samples. Samples processed immediately are marked t=0 and temp=NA. Processing delay is indicated for 4 hour (t=4) or 24 hours (t=24). Holding temperature is indicated for 4°C (temp=4C), 20°C (temp=20C), or 37°C (temp=37C). Missing values due to lack of blood volume are identified with an 'NA'.

participant #	capillary	venous	venous	capillary	venous	venous	venous	venous	venous	venous	venous	venous	venous	venous	venous	venous	venous	venous
	BD	BD	BD	Sarstedt	Sarstedt	Sarstedt	BD	BD	BD	BD	BD	BD	BD	BD	BD	BD	BD	BD
	plasma	plasma	serum	plasma	plasma	serum	plasma	plasma	plasma	serum	serum	serum	plasma	plasma	plasma	serum	serum	serum
	t=0	t=0	t=0	t=0	t=0	t=0	t=4	t=4	t=4	t=4	t=4	t=4	t=24	t=24	t=24	t=24	t=24	t=24
	temp=NA	temp=NA	temp=NA	temp=NA	temp=NA	temp=NA	temp=4C	temp=20C	temp=37C	temp=4C	temp=20C	temp=37C	temp=4C	temp=20C	temp=37C	temp=4C	temp=20C	temp=37C
1	0.968	0.802	0.856	NA	0.876	0.821	0.813	0.803	0.868	0.851	0.880	0.920	0.562	0.552	0.558	0.594	0.617	0.670
	0.933	0.786	0.854	NA	0.784	0.810	0.822	0.840	0.862	0.840	0.887	0.918	0.544	0.553	0.556	0.602	0.617	0.644
	0.905	0.816	0.841	NA	0.789	0.814	0.804	0.823	0.852	0.872	0.903	0.932	0.553	0.572	0.579	0.604	0.613	0.630
2	0.694	0.552	0.570	NA	0.531	0.665	0.511	0.534	0.585	0.568	0.607	0.578	0.810	0.819	0.845	0.858	0.905	0.904
	0.691	0.553	0.594	NA	0.532	0.547	0.541	0.538	0.604	0.595	0.621	0.573	0.823	0.828	0.835	0.860	0.892	1.083
	NA	0.551	0.586	NA	0.520	0.584	0.528	0.527	0.575	0.570	0.611	0.585	0.807	0.808	0.852	0.853	0.921	0.884
3	0.621	0.542	0.559	NA	0.531	0.667	0.500	0.490	0.563	0.554	0.599	0.567	0.500	0.657	0.500	0.589	0.581	0.597
	0.609	0.504	0.562	NA	0.525	0.545	0.503	0.519	0.540	0.564	0.595	0.569	0.508	0.506	0.502	0.593	0.503	0.598
	0.656	0.495	0.548	NA	0.536	0.539	0.503	0.504	0.541	0.586	0.590	0.578	0.509	0.485	0.596	0.601	0.601	0.575
4	0.677	0.610	0.683	NA	0.575	0.655	0.622	0.613	0.681	0.668	0.735	0.716	0.603	0.612	0.657	0.662	0.667	0.670
	0.676	0.623	0.658	NA	0.603	0.663	0.622	0.611	0.682	0.665	0.703	0.717	0.599	0.603	0.639	0.770	0.689	0.763
	0.671	0.609	0.664	NA	0.608	0.662	0.618	0.629	0.675	0.664	0.716	0.715	0.612	0.619	0.646	0.673	0.671	0.684
5	0.637	0.599	0.610	NA	0.557	0.602	0.580	0.618	0.609	0.638	0.685	0.657	0.582	0.607	0.602	0.636	0.628	0.646
	0.600	0.600	0.600	NA	0.558	0.382	0.578	0.608	0.616	0.645	0.668	0.659	0.588	0.582	0.653	0.679	0.629	0.646
	NA	0.596	0.606	NA	0.572	0.598	0.576	0.604	0.612	0.621	0.668	0.670	0.571	0.581	0.590	0.650	0.638	0.644
6	0.678	0.585	0.638	NA	0.789	0.591	NA	NA	NA	0.593	0.652	0.624	NA	NA	NA	0.604	0.657	0.618
	0.685	0.576	0.619	NA	0.578	0.598	NA	NA	NA	0.620	0.651	0.622	NA	NA	NA	0.602	0.637	0.673
	NA	0.577	0.626	NA	0.557	0.601	NA	NA	NA	0.610	0.657	0.606	NA	NA	NA	0.633	0.634	0.609
7	0.790	0.641	0.690	NA	0.604	0.655	0.577	0.591	0.630	0.619	0.670	NA	0.654	0.600	0.617	0.656	0.650	0.609
	0.743	0.642	0.691	NA	0.648	0.640	0.578	0.601	0.648	0.611	0.668	0.637	0.596	0.686	0.642	0.642	0.647	0.607
	NA	0.688	0.687	NA	0.622	0.641	0.578	0.598	0.649	0.618	0.680	0.629	0.585	0.594	0.611	0.656	0.641	0.616
8	0.662	0.634	0.721	NA	0.625	0.677	0.560	0.679	0.622	0.605	0.644	0.630	0.670	0.624	0.606	0.632	0.619	0.755
	0.695	0.635	0.650	NA	0.624	0.648	0.588	0.672	0.627	0.607	0.677	0.633	0.603	0.583	0.581	0.630	0.616	0.614
	0.744	0.638	0.708	NA	0.632	0.749	0.580	0.663	0.627	0.589	0.640	0.635	0.586	0.565	0.587	0.631	0.690	0.602
9	0.726	0.736	0.794	NA	0.735	0.739	0.686	0.661	0.696	0.695	0.731	0.679	0.708	0.696	0.679	0.700	0.707	0.669
	0.743	0.746	0.768	NA	0.727	0.741	0.667	0.673	0.705	0.703	0.797	0.679	0.681	0.689	0.686	0.709	0.709	0.673
	0.731	0.727	0.802	NA	0.719	0.742	0.664	0.661	0.700	0.710	0.731	0.673	0.685	0.688	0.676	0.696	0.706	0.673
10	0.550	0.570	0.559	NA	0.591	0.584	0.549	0.547	0.571	0.561	0.582	0.575	0.526	0.518	0.549	0.592	0.576	0.593
	0.523	0.584	0.568	NA	0.573	0.577	0.574	0.556	0.565	0.563	0.581	0.597	0.539	0.547	0.555	0.628	0.583	0.582

	0.527	0.579	0.580	NA	0.590	0.596	0.524	0.540	0.571	0.564	0.578	0.582	0.540	0.553	0.567	0.594	0.605	0.618
	0.681	0.665	0.738	NA	0.717	0.782	0.632	0.642	0.723	0.700	0.741	0.710	0.689	0.661	0.802	0.736	0.751	0.743
**11**	0.713	0.659	0.722	NA	0.729	0.757	0.642	0.653	0.689	0.712	0.741	0.692	0.685	0.652	0.691	0.744	0.748	0.721
	0.684	0.718	0.743	NA	0.733	0.745	0.612	0.653	0.688	0.705	0.722	0.693	0.656	0.656	0.691	0.773	0.730	0.727
	0.898	0.814	0.818	NA	0.779	0.818	0.766	0.782	0.796	0.798	0.843	0.763	0.756	0.762	0.763	0.853	0.854	0.799
**12**	0.888	0.814	0.814	NA	0.777	0.802	0.774	0.778	0.792	0.808	0.837	0.784	0.871	0.741	0.770	0.821	0.891	0.856
	0.896	0.775	0.821	NA	0.821	0.814	0.768	0.747	0.810	0.802	0.836	0.783	0.760	NA	0.777	0.816	0.836	0.821
	0.671	0.607	0.645	NA	0.656	0.809	0.579	0.618	0.616	0.592	0.651	0.600	0.602	0.612	0.627	0.619	0.644	0.671
**13**	0.635	0.630	0.620	NA	0.625	0.634	0.584	0.601	0.613	0.576	0.634	0.647	0.588	0.608	0.659	0.625	0.633	0.662
	0.617	0.612	0.672	NA	0.653	0.686	0.609	0.603	0.642	0.583	0.638	0.683	0.595	0.599	0.620	0.611	0.644	0.602
	0.706	0.609	0.702	NA	0.613	0.707	0.631	0.659	0.704	0.713	0.769	0.684	0.656	0.657	0.730	0.748	0.745	0.696
**14**	0.736	0.617	0.643	NA	0.616	0.697	0.682	0.687	0.792	0.718	0.775	0.682	0.645	0.657	0.984	0.717	0.751	0.691
	0.709	0.650	0.622	NA	0.623	0.699	0.651	0.656	0.703	0.704	0.778	0.686	0.751	0.658	0.736	NA	0.754	0.712
	0.750	0.734	0.763	NA	0.686	0.706	0.778	0.739	0.765	0.752	0.773	0.726	0.707	0.716	0.749	0.793	0.786	0.740
**15**	0.727	0.737	0.734	NA	0.698	0.709	0.746	0.724	0.774	0.736	0.785	0.725	0.688	0.719	0.760	0.803	0.775	0.728
	0.737	0.633	0.745	NA	0.678	0.712	0.724	0.729	0.790	0.750	0.786	0.743	0.689	0.728	0.774	0.784	0.768	0.778
	0.637	0.549	0.551	NA	0.539	0.567	0.513	0.528	0.548	0.584	0.647	0.570	0.567	0.595	0.588	0.518	0.540	0.581
**16**	0.567	0.539	0.553	NA	0.531	0.552	0.511	0.533	0.583	0.591	0.632	0.577	0.575	0.599	0.581	0.523	0.534	0.639
	0.571	0.530	0.584	NA	0.523	0.541	0.523	0.517	0.852	0.573	0.627	0.569	0.585	0.617	0.605	0.534	0.528	0.587
	0.914	0.850	0.928	NA	0.898	1.005	0.938	0.911	0.964	0.938	1.019	0.982	0.958	0.994	0.927	0.998	0.925	NA
**17**	0.933	0.855	0.933	NA	0.893	0.936	0.928	0.889	0.951	0.936	1.019	0.937	0.976	1.004	0.944	0.913	0.935	1.016
	0.899	0.839	0.931	NA	0.899	0.956	0.867	0.922	0.960	0.930	1.013	1.135	0.975	1.012	0.988	0.936	0.924	1.035
	0.787	0.578	0.565	NA	0.566	0.591	0.546	0.574	0.602	0.641	0.719	0.808	0.546	0.562	0.668	0.679	0.682	0.676
**18**	0.836	0.563	0.568	NA	0.570	0.591	0.538	0.592	0.601	0.619	0.705	0.683	0.534	0.573	0.622	0.680	0.688	0.654
	0.839	0.574	0.565	NA	0.572	0.600	0.562	0.567	0.590	0.617	0.692	0.677	0.536	0.562	0.628	0.665	0.690	0.659
	0.568	0.521	0.561	NA	0.540	0.536	0.579	0.610	0.604	0.644	0.672	0.626	0.585	0.616	0.691	0.657	0.726	0.629
**19**	NA	0.590	0.534	NA	0.544	0.564	0.611	0.609	0.612	0.638	0.688	0.619	0.603	0.600	0.704	0.663	0.658	0.805
	0.583	0.506	0.510	NA	0.522	0.530	0.588	0.701	0.625	0.642	0.683	0.637	0.597	0.601	0.668	0.632	0.668	0.646
	0.866	0.763	0.814	NA	0.726	0.771	0.821	0.852	0.846	0.856	0.910	0.893	0.814	0.817	0.902	0.919	0.993	0.986
**20**	0.853	0.735	0.796	NA	0.728	0.769	0.837	0.818	0.854	0.854	0.926	0.997	0.809	0.805	0.875	0.912	0.999	0.985
	0.867	0.749	0.794	NA	0.732	0.880	0.810	0.831	0.858	0.858	0.912	0.891	0.820	0.813	1.105	0.967	1.016	0.891
	0.712	0.640	0.655	NA	0.623	0.660	0.821	0.690	0.733	0.709	0.740	0.700	0.719	0.712	0.759	0.746	0.787	0.749
**21**	0.744	0.658	0.654	NA	0.686	0.648	0.700	0.721	0.735	0.717	0.751	0.695	0.706	0.698	0.754	0.787	0.781	0.726
	0.718	0.674	0.639	NA	0.623	0.664	0.788	0.704	0.726	0.718	0.746	0.726	0.706	0.701	0.753	0.733	0.773	0.729
	0.549	0.571	NA	NA	0.500	0.504	0.555	0.556	0.638	0.597	0.586	0.578	0.572	0.578	0.622	0.658	0.655	0.600
**22**	0.494	0.501	NA	NA	0.872	0.524	0.565	0.555	0.642	0.608	0.570	0.570	0.577	0.601	0.603	0.599	0.630	0.617
	0.557	0.542	NA	NA	0.488	0.503	0.555	0.556	0.624	0.612	0.576	0.574	0.572	0.586	0.603	0.611	0.630	0.612
	0.628	0.589	0.577	NA	0.605	0.579	0.625	0.668	0.673	0.655	0.752	0.723	0.645	0.649	0.668	0.690	0.735	0.670
**23**	0.532	0.576	0.568	NA	0.586	0.693	0.628	0.662	0.644	0.651	0.711	0.706	0.625	0.641	0.659	0.697	0.750	0.665
	0.525	0.621	0.572	NA	0.592	0.613	0.618	0.667	0.646	0.651	0.716	0.680	0.622	0.647	0.659	0.729	0.727	0.661
	0.813	0.691	0.645	NA	0.646	0.651	0.738	0.726	0.753	0.765	0.812	0.699	0.703	0.711	0.731	0.774	0.787	0.752
**24**	0.676	0.678	0.629	NA	0.636	0.647	0.746	0.706	0.793	0.722	0.769	0.702	0.714	0.741	0.734	0.744	0.765	0.741
	0.678	0.673	0.629	NA	0.647	0.648	0.725	0.702	0.781	0.747	0.773	0.725	0.712	0.715	0.760	0.745	0.786	0.743

	0.756	0.747	0.786	NA	0.711	0.755	0.867	0.739	0.812	0.795	0.878	0.829	0.737	0.868	0.824	0.752	0.798	0.800
**25**	0.753	0.763	0.766	NA	0.706	1.019	0.758	0.764	0.801	0.863	0.859	0.827	0.809	0.766	0.782	0.807	0.869	0.819
	0.781	0.756	0.770	NA	0.727	0.741	0.762	0.766	0.800	0.796	0.854	0.821	0.782	0.752	0.774	0.799	0.853	0.825
	0.677	0.629	0.639	0.632	0.603	0.628	0.629	0.648	0.671	0.761	0.743	0.697	0.642	0.638	0.680	0.693	0.720	0.677
**26**	0.746	0.623	0.658	0.596	0.590	0.616	0.613	0.664	0.703	0.677	0.732	0.673	0.632	0.649	0.650	0.723	0.716	0.689
	0.668	0.619	0.637	NA	0.617	0.615	0.665	0.635	0.682	0.668	0.728	0.685	0.641	0.650	0.659	0.693	0.720	0.681
	0.801	0.752	0.802	0.815	0.741	0.756	0.748	0.778	0.843	0.793	0.858	0.779	0.762	0.823	0.833	0.831	0.862	0.776
**27**	0.798	0.737	0.799	0.774	0.759	0.768	0.750	0.768	0.857	0.794	0.861	0.801	0.761	0.815	0.976	0.814	0.879	0.804
	0.805	0.733	0.799	NA	0.769	0.740	0.771	0.791	0.821	0.805	0.850	0.767	0.764	0.806	0.805	0.828	0.862	0.795
	0.717	0.704	0.721	0.709	0.674	0.725	0.713	0.710	0.789	0.818	0.828	0.721	0.716	0.724	0.778	0.778	0.828	0.754
**28**	0.743	0.686	0.722	0.718	0.670	0.710	0.708	0.720	0.809	0.757	0.808	0.747	0.724	0.732	0.797	0.770	0.834	0.746
	NA	0.675	0.723	NA	0.681	0.703	0.712	0.716	0.917	0.751	0.815	0.717	0.714	0.743	0.769	0.786	0.823	0.767
	0.697	0.674	0.698	NA	0.672	0.679	0.713	0.733	0.799	0.707	0.790	0.759	0.732	0.730	0.760	0.759	0.812	0.758
**29**	0.714	0.663	0.727	NA	NA	0.676	0.724	0.801	0.800	0.744	0.784	0.751	0.724	0.738	0.768	0.755	0.814	0.776
	0.688	0.655	0.716	NA	0.739	0.689	0.711	0.730	0.769	0.723	0.785	0.755	0.726	0.733	0.769	0.758	0.795	0.769
	0.649	0.607	NA	0.615	0.600	0.601	0.619	0.631	0.699	0.653	0.700	0.626	0.605	0.623	0.642	0.643	0.662	0.635
**30**	0.636	0.608	NA	0.625	0.591	0.608	0.617	0.636	0.709	0.667	0.700	0.615	0.588	0.619	0.623	0.633	0.675	0.625
	0.645	0.607	NA	0.615	0.603	0.619	0.621	0.628	0.712	0.674	0.699	0.621	0.592	0.610	0.627	0.655	0.656	0.629
	0.564	0.605	0.620	0.533	0.546	0.577	0.574	0.549	0.588	0.624	0.680	0.656	0.580	0.583	0.611	0.663	0.790	0.676
**31**	0.575	0.584	0.674	0.547	0.553	0.587	0.569	0.551	0.590	0.614	0.675	0.633	0.582	0.561	0.614	0.663	0.711	0.692
	0.649	0.592	0.609	NA	0.560	0.574	0.577	0.554	0.595	0.637	0.695	0.652	0.578	0.578	0.619	0.660	0.753	0.664
	0.796	0.744	0.761	0.712	0.701	0.711	0.803	0.790	0.766	0.762	0.796	0.843	0.749	0.761	0.782	0.775	0.847	0.791
**32**	0.802	0.755	0.770	0.724	0.868	0.714	0.787	0.739	0.777	0.759	0.792	0.789	0.751	0.769	0.760	0.761	0.800	0.787
	0.814	0.735	0.767	0.713	0.697	0.711	0.745	0.744	0.781	0.751	0.790	0.788	0.756	0.747	0.765	0.780	0.812	0.833
	0.652	0.549	0.594	NA	0.526	0.557	0.596	0.617	0.632	0.637	0.669	0.629	0.607	0.638	0.616	0.686	0.680	0.644
**33**	0.618	0.563	0.594	NA	0.531	0.560	0.608	0.624	0.648	0.631	0.673	0.618	0.593	0.600	0.638	0.652	0.708	0.623
	0.653	0.567	0.607	NA	0.535	0.617	0.621	0.620	0.643	0.648	0.694	0.624	0.580	0.597	0.637	0.692	0.671	0.634
	NA	0.602	0.629	NA	0.578	0.564	0.648	0.649	0.691	0.661	0.703	0.711	0.679	0.653	0.674	0.676	0.705	0.689
**34**	NA	0.601	0.614	NA	0.578	0.564	0.665	0.667	0.673	0.641	0.682	0.685	0.658	0.644	0.681	0.663	0.690	0.674
	NA	0.593	0.611	NA	0.570	0.587	0.626	0.649	0.668	0.657	0.678	0.676	0.697	0.650	0.689	0.685	0.720	0.702
	0.743	0.750	0.785	0.707	0.699	0.730	0.777	0.755	0.806	0.851	0.911	0.877	0.803	0.810	0.800	0.953	0.919	0.866
**35**	0.746	0.748	0.806	0.700	0.691	0.733	0.787	0.799	0.784	0.854	0.905	0.884	0.774	0.797	0.815	0.881	0.952	0.859
	0.743	0.789	0.781	0.769	0.696	0.735	0.747	0.756	0.799	0.864	0.920	0.879	0.827	0.800	0.802	0.889	0.914	0.857
	0.808	0.774	0.792	NA	0.731	0.765	0.773	0.780	0.807	0.819	0.965	0.888	0.827	0.818	0.876	0.847	0.901	0.873
**36**	0.801	0.765	0.797	NA	0.720	0.754	0.762	0.786	0.813	0.839	0.885	0.862	0.798	0.820	0.843	0.861	0.904	0.879
	0.802	0.763	0.799	NA	0.734	0.783	0.790	0.796	0.806	0.834	0.894	0.866	0.798	0.879	0.842	0.897	0.902	0.866
	0.680	0.591	0.646	NA	0.570	0.595	0.715	0.655	0.726	0.651	0.776	0.704	0.617	0.688	0.835	0.747	0.796	0.738
**37**	0.670	0.592	0.631	NA	0.562	0.579	0.630	0.652	0.713	0.660	0.775	0.700	0.692	0.686	0.722	0.751	0.789	0.745
	0.682	0.606	0.633	NA	0.560	0.590	0.698	0.664	0.739	0.655	0.752	0.694	0.623	0.725	0.723	0.749	0.884	0.742
	0.651	0.654	0.674	0.601	0.609	0.651	0.698	0.675	0.726	0.686	0.753	0.777	0.687	0.694	0.724	0.772	0.787	0.756
**38**	0.656	0.639	0.705	0.606	0.621	0.633	0.683	0.683	0.717	NA	0.755	0.741	0.686	0.691	0.774	0.753	0.783	0.747
	0.659	0.641	0.685	0.588	0.600	0.662	0.667	0.673	1.011	0.691	0.744	0.734	0.686	0.760	NA	0.752	0.858	0.756
**39**	0.837	0.787	0.804	NA	0.732	0.756	0.813	0.822	0.887	0.840	0.902	0.817	0.811	0.814	0.902	0.897	0.914	0.817

	0.826	0.775	0.806	NA	0.729	0.768	0.783	0.810	0.896	0.866	0.909	0.821	0.808	0.811	0.883	0.877	0.926	0.814
	0.840	0.767	0.815	NA	0.745	0.752	0.895	0.799	0.874	0.845	0.909	0.810	0.812	0.975	0.868	0.893	0.923	0.898
**40**	0.793	0.704	0.761	NA	0.673	0.706	0.747	0.758	0.809	0.778	0.811	0.768	0.752	0.754	0.823	0.812	0.834	0.800
	0.809	0.702	0.743	NA	0.678	0.697	0.756	0.780	0.793	0.810	0.867	0.783	0.740	0.745	0.828	0.811	0.836	0.802
	0.798	0.700	0.754	NA	0.690	0.680	0.775	0.751	0.791	0.790	0.821	0.773	0.799	0.761	0.820	0.813	0.828	0.812
**41**	0.694	0.610	0.594	NA	0.614	0.622	NA	NA	NA	0.780	0.727	0.707	NA	NA	NA	0.708	0.736	0.756
	0.695	0.641	0.645	NA	0.614	0.636	NA	NA	NA	0.711	0.722	0.693	NA	NA	NA	0.705	0.745	0.739
	0.703	0.617	0.650	NA	0.619	0.630	NA	NA	NA	0.704	0.737	0.718	NA	NA	NA	0.706	0.717	0.723
**42**	0.773	0.737	0.761	NA	0.715	0.760	0.782	0.821	0.821	0.854	0.851	0.825	0.774	0.794	0.840	0.838	0.882	0.812
	0.770	0.765	0.737	NA	0.715	0.751	0.799	0.824	0.830	0.849	0.848	0.822	0.783	0.792	0.818	0.838	0.884	0.834
	0.779	0.742	0.764	NA	0.716	0.729	0.799	0.854	0.816	0.859	0.906	0.831	0.827	0.793	0.817	0.830	0.886	0.827
**43**	NA	0.714	0.744	NA	0.753	0.726	0.775	0.783	0.838	0.835	0.860	0.844	0.767	0.800	0.787	0.856	0.870	0.821
	NA	0.709	0.752	NA	0.713	0.711	0.901	0.791	0.849	0.830	0.863	0.827	0.932	0.765	0.801	0.845	0.870	0.813
	NA	0.745	NA	NA	0.716	0.718	0.953	0.795	0.835	0.850	0.863	0.843	0.781	0.786	0.791	0.873	0.855	1.011
**44**	0.569	0.535	0.585	NA	0.551	0.569	0.610	0.623	0.620	0.624	0.687	0.650	0.612	0.587	NA	0.668	0.665	0.648
	0.562	0.543	0.591	NA	0.546	0.577	0.593	0.615	0.635	0.628	0.686	0.648	0.594	0.588	NA	0.660	0.667	0.643
	0.565	0.546	0.595	NA	0.581	0.566	0.589	0.600	0.631	0.630	0.669	0.650	0.631	0.614	NA	0.658	0.662	0.668
**45**	0.565	NA	0.546	NA	0.606	0.547	0.605	0.628	0.654	0.612	0.677	0.641	0.596	0.628	0.626	0.632	0.693	0.648
	0.561	0.525	NA	NA	0.533	0.553	NA	0.613	0.654	0.621	0.662	0.791	0.580	0.632	0.626	0.634	0.679	0.655
	0.640	0.529	0.560	NA	0.540	0.549	0.651	0.647	0.645	0.607	0.711	0.604	0.622	0.613	0.639	0.622	0.679	0.668
**46**	0.804	0.712	0.769	0.729	0.746	0.746	0.775	0.787	0.839	0.814	0.862	0.823	0.856	0.811	0.823	0.861	0.973	0.837
	0.725	0.706	0.768	0.728	0.799	0.734	0.769	0.782	0.851	0.815	0.864	0.802	0.810	0.817	0.805	0.839	0.885	0.828
	0.736	0.702	0.811	0.742	0.736	0.737	0.772	0.833	0.843	0.832	0.859	0.809	0.795	NA	0.809	0.856	0.886	NA
**47**	0.676	0.656	0.704	NA	0.650	0.689	0.747	0.732	0.831	0.779	0.832	0.785	NA	NA	NA	0.823	1.041	0.796
	0.704	0.653	0.679	NA	0.684	0.683	0.755	0.749	0.856	0.777	0.830	0.786	NA	NA	NA	0.806	0.846	0.830
	NA	0.649	0.687	NA	0.683	0.677	0.751	0.940	0.851	NA	0.826	0.794	NA	NA	NA	0.794	0.853	0.795
**48**	0.746	0.703	0.817	0.809	0.761	0.757	0.802	0.807	0.829	0.880	0.951	0.922	0.828	0.844	0.852	0.993	0.968	0.896
	0.744	0.696	0.775	0.746	0.684	0.759	0.794	0.816	0.838	1.045	0.968	0.934	0.830	0.840	0.892	0.941	0.946	0.962
	0.755	0.693	0.773	0.732	0.694	0.761	0.793	0.817	0.826	0.927	0.959	0.947	0.832	0.865	0.855	0.967	0.993	0.920
**49**	0.679	0.615	0.608	0.639	0.637	0.625	0.775	0.760	0.808	0.797	0.777	0.815	0.776	0.756	0.793	0.799	0.865	0.835
	0.714	0.613	0.595	NA	0.643	0.639	0.732	0.761	0.773	0.780	NA	0.814	0.765	1.022	0.829	0.836	0.904	0.829
	0.712	0.602	0.595	NA	0.640	0.347	0.791	0.769	0.775	0.767	0.833	0.900	0.785	0.787	0.813	0.794	0.908	0.836
**50**	0.638	0.552	0.627	NA	0.556	0.751	0.737	0.737	0.808	0.807	0.888	0.892	0.747	0.761	0.790	0.827	0.903	0.866
	0.635	0.557	0.648	NA	0.546	0.742	0.729	0.752	0.759	0.791	0.882	0.877	0.711	0.738	0.797	0.855	0.909	0.845
	0.666	0.570	0.626	NA	0.582	0.655	0.754	0.724	0.757	0.800	0.912	0.855	0.733	0.741	0.802	0.844	0.942	0.849
**51**	0.717	0.622	0.655	0.680	0.629	0.678	0.730	0.714	0.774	0.800	0.841	0.784	0.886	0.773	0.729	0.928	0.879	0.774
	0.685	0.614	0.652	0.663	0.628	0.654	0.717	0.714	0.778	0.780	0.837	0.829	0.764	0.813	0.783	0.783	0.993	0.786
	0.670	0.658	0.734	NA	0.633	0.649	0.718	0.761	0.800	0.795	0.831	0.847	0.709	0.739	0.728	0.776	0.784	0.758
**52**	0.614	0.567	0.595	0.557	0.597	0.614	0.665	0.711	0.709	0.696	0.735	0.669	0.678	0.667	0.659	0.692	0.740	0.706
	0.584	0.567	0.583	0.539	0.593	0.622	0.674	0.670	0.784	0.700	0.737	0.672	0.683	0.666	0.684	0.682	0.698	0.699
	0.601	0.571	0.595	NA	0.610	0.626	0.651	0.738	0.745	0.677	0.695	0.704	0.665	0.667	0.660	0.766	0.707	0.704
**53**	0.665	0.641	0.637	0.618	0.619	0.647	NA	NA	NA	0.732	0.798	0.777	NA	NA	NA	0.700	0.761	0.739
	0.694	0.670	0.626	0.623	0.609	0.699	NA	NA	NA	0.728	0.800	0.961	NA	NA	NA	0.710	0.789	0.745

	0.660	0.642	0.621	0.617	0.594	0.636	NA	NA	NA	NA	0.790	0.780	NA	NA	NA	0.703	0.816	0.732
54	0.616	0.541	0.569	0.592	0.571	0.589	0.631	0.645	0.687	0.665	0.768	0.745	0.691	0.754	0.695	0.629	0.640	0.698
	0.716	0.673	0.582	0.592	0.562	0.592	0.617	0.657	0.680	0.649	0.745	0.867	0.706	0.723	0.710	0.627	0.649	0.681
	0.621	0.529	0.583	NA	0.571	0.591	0.601	0.689	0.675	0.695	0.762	0.712	0.693	0.733	0.704	0.618	0.639	0.669
55	0.707	0.744	0.729	0.758	0.694	0.750	0.747	0.747	0.788	0.834	0.861	0.857	0.738	0.749	0.747	0.842	0.879	0.827
	0.718	0.662	NA	0.721	0.696	0.747	0.742	0.735	0.762	0.843	0.862	0.900	0.750	0.758	0.765	0.835	0.873	0.849
	NA	0.688	NA	NA	0.682	0.766	0.735	0.738	0.774	0.819	0.843	0.807	0.733	0.736	0.766	0.849	0.886	0.844
56	0.666	0.604	0.627	0.600	0.640	0.666	0.668	0.688	0.702	0.679	0.748	0.714	0.674	0.718	0.711	0.733	0.781	0.740
	0.649	0.602	0.612	0.615	0.691	0.625	0.683	0.679	0.698	0.690	0.745	0.706	0.683	0.722	0.725	0.730	0.757	0.747
	0.656	0.598	0.624	NA	0.631	0.636	0.664	0.671	0.692	0.699	0.775	0.715	0.678	0.670	0.716	0.737	0.764	0.737
57	0.683	0.642	0.708	0.655	0.648	0.654	0.885	0.764	0.752	0.778	0.790	0.755	0.727	0.741	0.771	0.763	0.808	0.774
	0.846	0.748	0.680	0.688	0.662	0.668	0.761	0.748	0.773	0.764	0.784	0.771	0.737	0.762	0.773	0.787	0.786	0.738
	0.694	0.647	0.672	NA	0.640	0.670	0.719	0.747	0.828	0.758	0.802	0.756	0.735	0.726	NA	0.764	0.785	0.799
58	0.661	0.647	0.654	0.644	0.614	0.653	0.658	0.687	0.708	0.714	0.765	0.744	0.658	0.676	0.674	0.750	0.773	0.747
	0.658	0.611	0.651	0.676	0.625	0.635	0.676	0.658	0.699	0.712	0.742	0.746	0.659	0.696	0.703	0.709	0.775	0.736
	0.652	0.612	0.653	0.718	0.625	0.642	0.783	0.665	0.705	0.709	0.761	0.739	0.661	0.656	0.797	0.729	0.770	0.752
59	0.509	0.462	0.480	0.425	0.470	0.477	0.544	0.507	0.560	0.529	0.591	0.532	0.529	0.535	0.558	0.547	0.598	0.549
	0.511	0.458	0.485	0.478	0.457	0.472	0.487	0.643	0.551	0.517	0.600	0.552	0.496	0.518	0.564	0.560	0.601	0.568
	0.494	NA	0.507	0.469	0.462	0.670	0.485	0.501	0.554	0.531	0.618	0.523	0.503	0.509	0.559	0.550	0.611	0.545
60	NA	0.659	0.704	NA	0.678	0.677	0.708	0.812	0.728	0.743	0.786	0.880	0.734	0.753	0.724	0.774	0.809	0.875
	NA	0.768	0.711	NA	0.656	0.688	0.716	0.766	0.777	0.768	0.783	0.818	0.714	0.694	0.790	0.767	0.806	0.802
	NA	0.649	0.724	NA	0.707	0.674	0.696	0.694	0.737	0.786	0.794	0.745	0.920	0.716	0.799	0.797	0.788	0.791
